# Immunogenetics, sylvatic plague and its vectors: insights from the pathogen reservoir *Mastomys natalensis* in Tanzania

**DOI:** 10.1007/s00251-023-01323-7

**Published:** 2023-10-19

**Authors:** Lavinia Haikukutu, Japhet R. Lyaku, Charles M. Lyimo, Seth J. Eiseb, Rhodes H. Makundi, Ayodeji Olayemi, Kerstin Wilhelm, Nadine Müller-Klein, Dominik W. Schmid, Ramona Fleischer, Simone Sommer

**Affiliations:** 1 Department of Wildlife Management, Sokoine University of Agriculture, Morogoro, Tanzania; 2 Institute of Evolutionary Ecology and Conservation Genomics, University of Ulm, Ulm, Germany; 3 Department of Paraclinical Studies, School of Veterinary Medicine, University of Namibia, Windhoek, Namibia; 4 Department of Animal, Aquaculture and Range Sciences, Sokoine University of Agriculture, Chuo Kikuu, Morogoro, Tanzania; 5 Department of Environmental Sciences, University of Namibia, Windhoek, Namibia; 6 Africa Centre of Excellence for Innovative Rodent Pest Management and Biosensor Technology Development, Sokoine University of Agriculture, Morogoro, Tanzania; 7 Natural History Museum, Obafemi Awolowo University, Ile Ife, Osun State Nigeria

**Keywords:** MHC, *Mastomys natalensis*, *Yersinia pestis*, Plague, Pathogen resistance, Fleas, Tanzania

## Abstract

*Yersinia pestis* is a historically important vector-borne pathogen causing plague in humans and other mammals. Contemporary zoonotic infections with *Y. pestis* still occur in sub-Saharan Africa, including Tanzania and Madagascar, but receive relatively little attention. Thus, the role of wildlife reservoirs in maintaining sylvatic plague and spillover risks to humans is largely unknown. The multimammate rodent *Mastomys natalensis* is the most abundant and widespread rodent in peri-domestic areas in Tanzania, where it plays a major role as a *Y. pestis* reservoir in endemic foci. Yet, how *M. natalensis*’ immunogenetics contributes to the maintenance of plague has not been investigated to date. Here, we surveyed wild *M. natalensis* for *Y. pestis* vectors, i.e., fleas, and tested for the presence of antibodies against *Y. pestis* using enzyme-linked immunosorbent assays (ELISA) in areas known to be endemic or without previous records of *Y. pestis* in Tanzania. We characterized the allelic and functional (i.e., supertype) diversity of the major histocompatibility complex (MHC class II) of *M. natalensis* and investigated links to *Y. pestis* vectors and infections. We detected antibodies against *Y. pestis* in rodents inhabiting both endemic areas and areas considered non-endemic. Of the 111 nucleotide MHC alleles, only DRB*016 was associated with an increased infestation with the flea *Xenopsylla*. Surprisingly, we found no link between MHC alleles or supertypes and antibodies of *Y. pestis*. Our findings hint, however, at local adaptations towards *Y. pestis* vectors, an observation that more exhaustive sampling could unwind in the future.

**Supplementary Information:**

The online version contains supplementary material available at 10.1007/s00251-023-01323-7.

## Introduction

Plague, a bacterial disease caused by *Yersinia pestis* is notorious for causing one of mankind’s worst pandemics, referred to as “The Black Death,” that wiped out approximately 50 million people in Afro-Eurasia between 1346 and 1350 (Benedictow 2004; Klunk et al. 2022). In modern days, plague occurs sporadically in a few countries around the world, earning it the badge of a re-emerging, yet neglected zoonotic disease (Ditchburn and Hodgkins 2019; Eisen et al. 2021). Primarily a disease of small mammals, plague latently circulates among rodent hosts (and sometimes shrews) via flea vectors and usually goes unnoticed until a spillover to humans occurs (Makundi et al. 2008), which can lead to human-to-human transmission. Human plague reports form the main available source of worldwide *Y. pestis* distribution (Stenseth et al. 2008) but are imperfect tools for mapping the distribution of the disease given its quiescent nature. Furthermore, plague is usually not maintained in humans but in wildlife, thus, comprehensive records of wildlife plague sero-prevalence can identify new hotspots (Kilonzo et al. 2005) and may be used to predict spillover risk.

Although perceived by many as an ancient disease, plague re-emerges as a threat to human health in Tanzania, where spillover to humans occasionally results in deadly outbreaks in endemic foci. Introduced from Uganda in 1883, the disease spread to other parts of the country through slave and ivory caravans and established foci on these ancient routes (Msangi 1968; Kilonzo et al. 2005). The first confirmed record of a plague epidemic occurred in Tanzania in 1886 in Image, Iringa. Outbreaks foreshadowed by high rat mortality and heavy rains, occurred in several localities in Iringa until 1937 (Koch 1898; Msangi 1968). Moreover, plague outbreaks occurred in Mbulu in 1904, though this was only confirmed microbiologically in 1917 (Kilonzo and Mtoi 1983; Msangi 1968). The deadliest outbreak ever recorded in the country occurred in Lushoto, with nearly 8000 cases and 640 deaths between 1980 and 2004 (Kilonzo and Mhina 1982; Ziwa et al. 2013a, b). The most recent epidemic occurred in 2007 in Mbulu (Makundi et al. 2008). Even though outbreaks in the country have faded over the years, sporadic cases are still being reported in the vicinity of Mbulu and in the neighboring district Babati (Mwalimu et al. 2022).

Numerous small wildlife species have been identified as potential reservoirs of plague in Tanzania, including the Natal multimammate mouse (*Mastomys natalensis*), the black rat (*Rattus rattus*), *Lophuromys* spp., the delectable soft-furred mouse (*Praomys delectorum*), the typical striped grass mouse (*Lemniscomys striatus*), the woodland dormouse (*Graphiurus murinus*), *Mus* sp., and *Crocidura* sp. (Kilonzo et al. 2005, 2006; Makundi et al. 2008; Ziwa et al. 2013b; Haule et al. 2014). Plague reservoir species differ in their susceptibility to *Y. pestis* infection as shown by experimental infections (Shepherd et al. 1986; Rahalison et al. 2004; Andrianaivoarimanana et al. 2018; Russell et al. 2019). What exactly mediates wildlife hosts susceptibility or resistance to *Y. pestis* infection is often entirely unknown, but host immunity is likely key. The major histocompatibility complex (MHC), a gene-dense region encoding glycoproteins that bind peptides (self and foreign) and present them to T-cells for recognition and initiation of T-cell responses, plays a major role in adaptive immunity of jawed vertebrates (Kaufman 2018). The classical MHC I initiate defenses against intracellular pathogens (i.e., viruses), whereas MHC II triggers immune reactions against extra-cellular parasites (i.e., bacteria and ectoparasites). Characterized by exceptional polymorphism (both in terms of allele number and sequence divergence), particularly at peptide binding sites, MHC genes have become an attractive model for studying pathogen-mediated selection (Sommer 2005; Spurgin and Richardson 2010) from the most basal jawed vertebrate lineages (e.g., Gaigher et al. 2023) to imperfectly (e.g., Gaczorek et al. 2023) or recently diverged species (e.g., Li et al. 2021; Bracamonte et al. 2022).

A central question in MHC research is how its polymorphism is maintained (Radwan et al. 2020). Aside from sexual selection, three non-exclusive mechanisms of pathogen-mediated selection are hypothesized to act on the identity, diversity, and frequency of MHC alleles: A diverse MHC allele repertoire and higher allelic divergence increase resistance to a diverse pool of parasites (i.e., heterozygote/divergent allele advantage), as, for example, reported for long-tailed giant rats (*Leopoldamys sabanus*, Lenz et al. 2009). Because pathogens evolve to circumvent detection by common MHC alleles, beneficial (often rare) MHC alleles become more frequent in the host population (i.e., negative frequency-dependent selection), as was found in Trinidadian Guppies (*Poecilia reticulata*, (Phillips et al. 2018). Lastly, distinct pathogen communities favor distinct immunogenetic profiles in geographically or temporally separated host populations (i.e., fluctuating selection), as seen in three-spined stickleback (*Gasterosteus aculeatus*, Eizaguirre et al. 2012). *Y. pestis* and its vectors also mediate selection on the MHC. In Gunnison’s prairie dog (*Cynomys gunnisoni*), a series of severe epizootic outbreaks of *Y. pestis* led to a near 100% decline in the population, and among the surviving population the most common MHC II DRB allele was experimentally linked to resistance against *Y. pestis* (Cobble et al. 2016). MHC heterozygous water voles (*Arvicola terrestris*) were co-infected by fewer fleas, mites, and ticks (Oliver et al. 2009). This implies a central role of MHC molecules in resistance to ectoparasitic vectors of *Y. pestis* and infections with *Y. pestis* itself.

In this study, we used the multimammate rat (*M. natalensis*) as a model system to investigate links between MHC II gene diversity and *Y. pestis* infection as well as flea burden to understand the century-long enigma of plague persistence in Tanzania. *M. natalensis* is a common rodent throughout sub-Saharan Africa and has been implicated in transmission of a range of zoonotic diseases including plague (Kilonzo and Mhina 1982), though local differences in susceptibility to plague exist across its range (Isaäcson et al. 1983; Shepherd et al. 1986). The species further harbors a range of ectoparasites (Makundi et al. 2008; Shilereyo et al. 2022), including key vectors, such as *Xenopsylla brasiliensis* and *Dinopsylus lypusus* which can be found in Tanzania (Msangi 1968; Kilonzo et al. 2006; Makundi et al. 2008; Gebrezgiher et al. 2023). Thus, *M. natalensis* is a likely candidate contributing to the transmission of plague to humans in Tanzania, but the underlying mechanisms are not well understood. Here, we carried out an enzyme-linked immunosorbent assays to assess *Y. pestis* status and recorded the ectoparasite (flea) burden on wild *M. natalensis* in selected sites with recent (Mbulu), past (over 20 years ago: Lushoto) and historic (> 100 years ago: Iringa and Kilolo) cases of human plague. Additionally, we tested whether flea infestation and *Y. pestis* infections were connected to rodents’ allelic and/or functional immunogenetic diversity.

## Materials and methods

### Study sites and rodent sampling

**Fig. 1:**
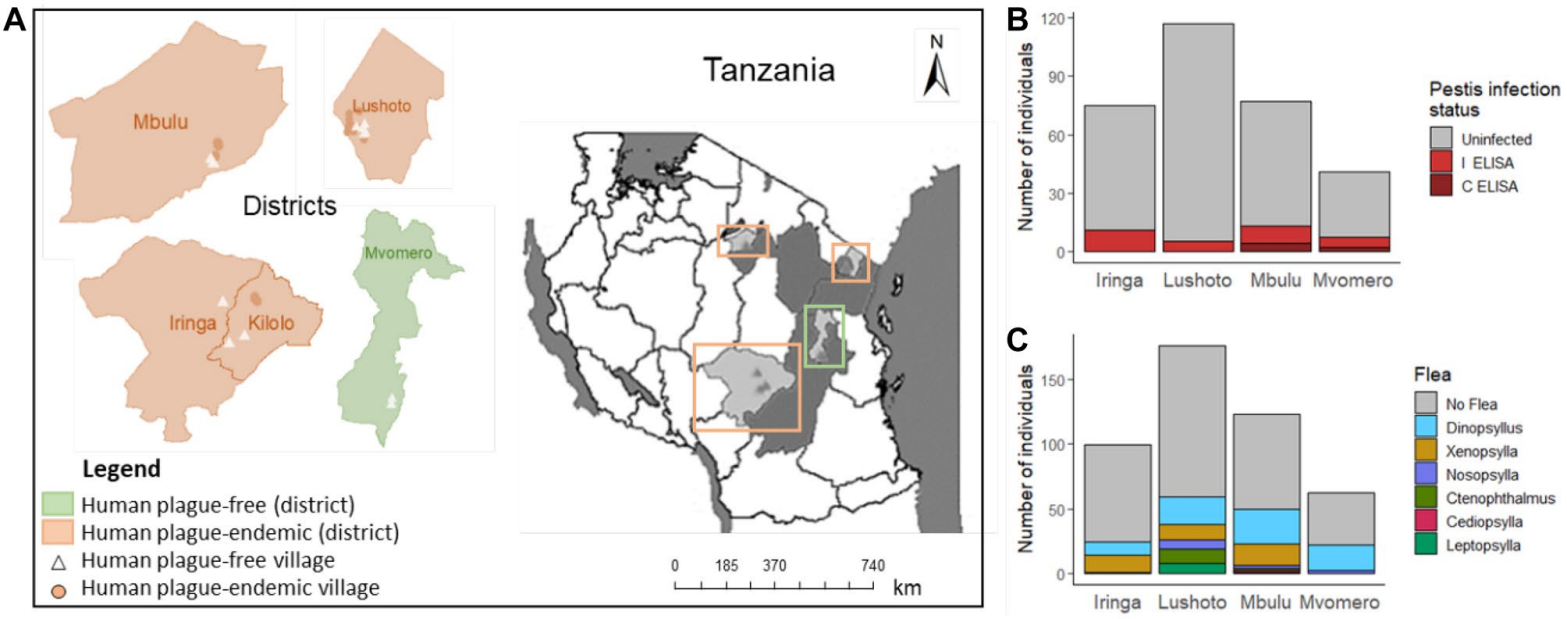
**A** Location of Tanzanian study sites where *M. natalensis* were captured. Orange-shaded areas are districts considered to be plague endemic, i.e., sites with a history of human plague, green indicates a non-plague district, i.e., site with no history of human plague. Villages sampled are depicted by white triangles (no human plague recorded) and orange circles (human plague recorded). **B** Indirect (i) and competitive (c) ELISA results for detection of antibodies against the fraction 1 antigen of *Y. pestis* in *M. natalensis* across the four sites sampled. **C** Number of individuals infected with different flea genera by site

Fieldwork was conducted between 2020 and 2021 in five districts: Iringa, Kilolo, Lushoto, Mbulu and Mvomero in Tanzania (Fig. 1A). These include areas with a history of human plague ((Iringa including parts of Kilolo district (*n* = 75), Lushoto (*n* = 117), Mbulu (*n* = 73)) and an area with no human plague history (Mvomero (*n* = 40)). Since the first plague records in a village called Image in Iringa region more than a century ago, restructuring of administrative divisions resulted in some villages affected by the plague epidemic no longer being part of Iringa district but are now part of Kilolo district. Hence, the two districts (Iringa and Kilolo) are hereinafter collectively referred to as “Iringa.” Sylvatic plague has never been investigated in Mvomero prior to this study, although the climatic and ecological conditions in Mvomero mirror those of plague endemic foci in Tanzania.

*Mastomys natalensis* individuals (*N* = 305) were live-captured in houses and crop fields using Sherman traps. Animals and their ectoparasites were anesthetized with diethyl ether and fleas were collected by thoroughly scouring the fur of the rodent with a small brush. The fleas were preserved in microtubes containing 70% ethanol and identified microscopically following taxonomic keys (Dunnet and Mardon 1974; Bahmanyar et al. 1976). Blood samples were collected by cardiac puncture and left at room temperature for serum separation. The separated serum was collected into sterile vials and stored at − 20 °C. Each rodent was sexed and weighed and an ear/tail tissue samples were taken and stored in 90% ethanol for DNA extraction.

### Serological detection of *Yersinia pestis*

The fraction 1 (F1) protein, a dominant surface antigen and an important virulence determinant of *Y. pestis* has been a target antigen for the development of many enzyme-linked immunosorbent assays (ELISA) (Rasoamanana et al. 1997; Choi et al. 2020; Hau et al. 2022). Protective antibodies against F1 antigen of *Y. pestis* (usually appearing between day 8 and day 13 after infection) have long been used in serological diagnosis of *Y. pestis* infection in both animals and humans (Meyer 1964; Shepherd et al. 1986; Esmaeili et al. 2023). Two ELISA tests were performed according to the ELISA protocol recommended by the Plague unit of Institute Pasteur in Madagascar (World Health Organization Collaborating Centre for Plague) with some modification (Rasoamanana et al. 1997; Dromigny et al. 1998). The first ELISA test aimed to detect IgG antibodies against the F1 antigen of *Y*. *pestis* and the second test was performed to determine the specificity of the antibodies detected.

Detection of immunoglobulin G (IgG) antibodies against F1 antigen of *Y. pestis* was conducted by indirect enzyme-linked immunosorbent assay (iELISA) as previously described (for details see Andrianaivoarimanana et al. 2012; Dromigny et al. 1998). The mean optical density (OD) obtained against the coating buffer alone was subtracted from the OD against F1 antigen (delta OD). In each plate, negative (3 sera) and positive sera from wild rodents were included as controls and sera were randomly scattered on the ELISA plates. For result interpretation, ratio (*R*) system was used, calculated as the ratio of the delta OD of the sample (OD of plate with serum sample—OD of plate with buffer only) to the mean delta OD of 3 negative sera + standard deviations (SD). Samples with an OD > 0.100 were considered positive. The OD thresholds were determined according to the best specificity and sensitivity (Youden’s index) from the receiver operating characteristic (ROC) curve and the conjugate used as described by Dromigny et al. (1998).

Samples with detectable anti-F1 antibodies were further subjected to the competitive blocking ELISA (cELISA) to determine the specificity (whether the antibodies detected were specific to *Y. pestis*) following a protocol previously described (Chu 2000). The two tests vary in sensitivity and specificity, while iELISA is more sensitive but less specific, cELISA is more specific but less sensitive. The protocol involves inhibiting the antibody present in each positive sample with a diluted F1 antigen prior to the competitive blocking ELISA technique. The specificity of the reaction was demonstrated by a decrease of OD value according to the amount of F1 antigen added. In contrast, the reaction is considered as non-specific if the OD value remained the same, regardless of the quantity of F1 antigen added prior to the test.

### High-throughput MHC sequencing

Total DNA was extracted from tissues using the ZR Zymo kit (Zymo Research, USA) following the manufacturer’s protocol. A 171 bp long fragment (covering functionally important antigen binding and recognition sites) on exon 2 of MHC class II DRB was amplified using JS1/JS2 primers (5′-GAGTGTCATTTCTACAACGGGACG-3′/5′-GATCCCGTAGTTGTG TCTGCA-3′ (Schad et al. 2004). These primers have an exceptional ability to amplify MHC class II DRB exon 2 in different mammalian species and have since been used on several rodents (Froeschke and Sommer 2005; Lenz et al. 2009), shrews (Oppelt et al. 2010), lagomorphs (Smith et al. 2011), chiropterans (Fleischer et al. 2022), marsupials (Meyer-Lucht et al. 2010), and non-human primates worldwide (Huchard et al. 2012). One DRB locus in the striped mouse (*Rhabdomys pumilio*, Froeschke and Sommer 2005) and two loci were previously amplified in the long-tailed giant rat (*Leopoldamys sabanus*, Lenz et al. 2009) using single stranded conformation polymorphism (SSCP); hence, we expected to amplify two or more loci with NGS. PCR conditions and allele amplification were tested with a subset of six samples in triplicates on an Illumina test run. Finally, amplification was performed in a 10-μL reaction volume with 1.0 μL of DNA sample, 300 nM of each primer, 5.0 μL AmpliTaq Gold 360 Master Mix (Applied Biosystems, Germany), 10 μL GC enhancer, and 2.4 μL H_2_O. The cycling parameters were an initial denaturation step at 95 °C for 10 min, followed by 28 cycles of a 30-s denaturation at 95 °C, 30-s annealing at 55 °C, and 60-s elongation at 72 °C with a final elongation for 3 min at 72 °C. Amplicons were visualized on 1.5% agarose gels to verify the fragment size. For library preparations, the JS1/JS2 primers were fused with general adapters (CS1/CS2) (Acces Array™ System for Illumina Sequencing Systems, © Fluidigm, USA). Four additional random base pairs were added to each forward primer for optimization of cluster recognition during sequencing (forward primer: CS1-NNNN-JS1; reverse primer: CS2-JS2). In a second PCR, sequencing adapters and a unique 10 bp barcode were added. The 20-μL reaction volume consisted of a 2.5-μL product from the initial PCR, 4.0 μL © Fluidigm barcode primers, 10 μL AmpliTaq Gold 360 Master Mix, 1.0 μL GC enhancer, and 2.5 μL H_2_O. Cycling conditions consisted of an initial denaturation of 10 min at 95 °C, 8 cycles of a 30-s denaturation at 95 °C, annealing for 30 s at 60 °C, and elongation for 60 s at 72 °C. Final elongation at 72 °C was set to 3 min. Barcoded PCR products were cleaned with beads using the NucleoMag NGS cleanup and size select kit (Macherey–Nagel, Germany) and pooled at equimolar ratios and prepared for sequencing according to the Miseq Reagent Kit Preparation Guide (Illumina, USA). The libraries were sequenced with the Illumina v2 500-cycles kit on a MiSeq platform.

### MHC allele calling using the ACACIA pipeline

A total of 9,892,122 raw reads were generated to characterize allelic diversity at the MHC II DRB exon 2 genes. Raw data from Illumina MiSeq sequencing was analyzed and processed using the ACACIA pipeline (Gillingham et al. 2021). In brief, the FastQC tool first assessed the sequencing quality of the paired-end reads. The untrimmed paired-end reads were then merged using FLASH (Magoč and Salzberg 2011) with a minimum overlap set to 50 bp and a maximum overlap of 250 bp. The subsequent steps were performed to identify all artefacts. First, sequences considered low quality, i.e., with a Phred score value < 30 at more than 10% of nucleotide positions were removed. Sequences that did not contain complete primer sequences were also removed. Furthermore, chimeras were detected and expunged using VSEARCH (Rognes et al. 2016) with default settings in ACACIA. All remaining sequences were blasted against a locally built rodent MHC-DRB sequences to remove unrelated sequences. All retained high-quality sequences were aligned using the MAFFT algorithm (Katoh and Standley 2013). Allele calling was performed with a clustering algorithm, the OLIGOTYPING tool (Eren et al. 2013) based on Shannon entropy (with a cutoff of 0.2), hence making it possible to distinguish very similar alleles and to detect variable positions in contrast to noise attributed to sequencing errors (Menke et al. 2014). Finally, putative alleles with less than 10 reads and 1% reads within the sample were excluded, and only individuals with a minimum coverage of at least 8000 raw reads (before merging) were kept for downstream analyses. About 12% of the samples (*n* = 36) were duplicated to assess repeatability and identify possible amplification and sequencing biases. Negative controls on sequencing runs were clean (< 162 reads after merging).

### Identifying positively selected sites (PSSs) and MHC supertyping

The PSS are presumably part of or close to functionally relevant antigen-binding sites. Consequently, MHC molecules with distinct amino acids at PSSs are presumed to bind distinct antigens and represent functional diversity (Cohen 2002; Sepil et al. 2013; Schwensow et al. 2019). To identify PSSs, we examined positive selection using the HYPHY software on the Datamonkey public webserver (Pond and Frost 2005; Weaver et al. 2018) using complementary methods: FEL (fixed effects likelihood), SLAC (single-likelihood ancestor counting), FUBAR (fast unconstrained Bayesian approximation), and MEME (mixed effects model of evolution). In addition, we used the program PAML4 (phylogenetic analysis by maximum likelihood) (Yang 2007) available for the PAML-X graphical user interface (Xu and Yang 2013) and tested the models M1/M2 and M7/M8 to identify sites under positive selection. Finally, we considered 10 sites that were supported by at least four of the tested methods for further analyses (Table S1).

MHC alleles with functionally similar pathogen binding affinities based on shared amino acid motifs at positively selected sites can then be clustered together into MHC supertypes (e.g., Doytchinova et al. 2004; Sepil et al. 2013). The rationale is that grouping alleles into supertypes allows for greater statistical power to detect biologically meaningful links with pathogens and host fitness. We grouped MHC alleles into supertypes using the discriminant analysis of principal components implemented in the R package adegenet (Jombart et al. 2010) and identified the ideal number of clusters suggested by the Bayesian information criterion curve (Fig. S1 and Table S2).

### Statistical analysis

All statistical analyses were performed using RStudio version 4.1.3 (RStudio Team 2015). Flea count and number of positive *Y. pestis* infections were compared between study sites using a generalized linear model with Poisson and binomial distribution, respectively. Then, an analysis of similarity (ANOSIM) with Jaccard distance was used to compare MHC allele and supertype composition between study sites. We restricted the analyses to MHC alleles present in at least 5% and MHC supertypes present in at least 10% of the individuals in each site. Associations between specific MHC alleles/supertypes and *Y. pestis* infections or flea presence were identified using co-occurrence analysis as outlined in the *cooccur* R package (Veech 2014; Griffith et al. 2016). Positive associations between an MHC allele/supertypes and *Y. pestis* or any of its vectors is assumed when the observed co-occurrence is significantly higher than the expected co-occurrence and this suggests susceptibility. Conversely, a negative co-occurrence suggests resistance. To avoid reporting positive results based on covariance between alleles/supertypes, cases in which alleles/supertypes are positively or negatively associated with each other, the more frequent allele/supertype was assumed to be driving the effect (Råberg et al. 2022). Significant co-occurrence results were confirmed with generalized linear models (GLMs) with binomial error structure, using *Y. pestis* infection and flea infestation (presence or absence) as response variable and the identified MHC II allele/supertype, the number of alleles/supertypes per individual, and sex as explanatory variables separate per study site. We retained the explanatory variables only from the single model with the lowest AIC score using the function dredge() in the *MuMIn* R package (Bartoń 2022).

## Results

### Detection of anti-F1 antibodies against *Yersinia pestis*

Indirect ELISA identified 30 M*. natalensis* individuals with detectable *Y. pestis* anti-F1 IgG accounting for 9.8% of the sampled individuals (Fig. 1B). In contrast, cELISA only found six *M. natalensis* individuals, i.e., 1.9%, with specific anti-F1 antibodies against *Y. pestis*. Owing to the low sensitivity of cELISA samples and, hence, lack of statistical power, only iELISA results were considered in the co-occurrence analysis and any further analyses. Importantly, five *M. natalensis* individuals from Mvomero were positive for anti-F1 antibodies, suggesting that sylvatic plague is not limited to areas where human plague has been reported previously.

### Flea abundance

A total of 268 fleas were collected from all examined hosts and each individual carried an average of 0.88 (± 1.68 (standard deviation (SD)). Fleas belonged to 6 genera*: Dinopsyllus*, *Xenopsylla*, *Nosopsylla*, *Ctenophthalmus*, *Cediopsylla*, and *Leptopsylla* (Fig. 1C)*. Dinopsyllus*, a presumed plague vector, was the most abundant flea across all sites, while surprisingly *Xenopsylla* another presumed plague vector, was also found in all study sites except Mvomero, where human plague has never been reported. The combined totals for *Dinopsyllus* and *Xenopsylla* flea species accounted for 80.6% (*n* = 216) of fleas collected from hosts. Fleas of the genus *Ctenophthalmus* (*n* = 17), *Cediopsylla* (*n* = 1), *Leptopsylla* (*n* = 14), and *Nosopsylla* (*n* = 20) accounted for the remaining 19.4% of fleas collected. Subsequent analyses focused on the two most abundant flea vectors *Dinopsyllus* and *Xenopsylla*, which are the presumed main plague vectors in Tanzania. The abundance of the common flea genus *Dinopsyllus* differed between study sites except for the comparisons Mbulu–Mvomero and Iringa–Lushoto (Table S3). A significant difference in *Xenopsylla* abundance was observed between Mbulu and Lushoto (Table S3).

### MHC II allele composition of *Mastomys natalensis*

A total of 305 individuals were genotyped with a mean of 29,093 (± 5530 SD) reads per sample (range: 8141–50, 425) and an average of 23,707 (± 4612 SD) reads after quality filtering. Allele call repeatability was 98.6% among the 36 replicates. Overall, 113 unique MHC class II alleles were identified, although two (DRB*29 and DRB*83) were removed as they contained stop codons. The resulting 111 putatively functional nucleotide alleles were translated into 91 amino acids sequences with 57 codon sites (Fig. S2). The MHC alleles clustered into nine distinct supertypes (Fig. S1 and Table S2).

**Fig. 2:**
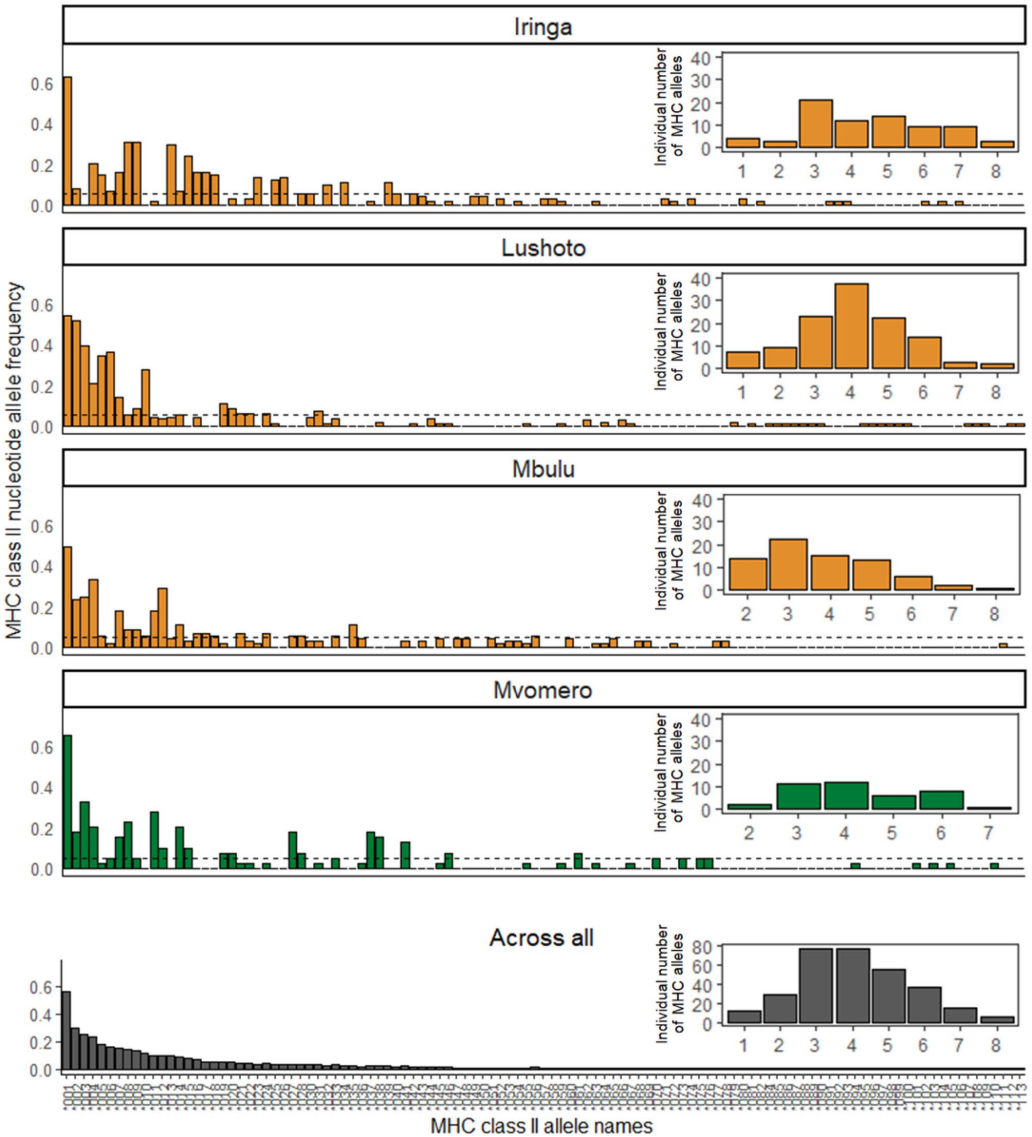
The frequency of MHC class II *DRB* alleles and total number of alleles (per individual among 305 M*. natalensis* genotyped) per site and across all sampling sites. Orange shows districts considered to be plague endemic, i.e., sites with a history of human plague, green indicates a non-plague district, i.e., site with no history of human plague. The dashed line indicates the 5% threshold for alleles to be included in further analyses

The total number of MHC II alleles per individual ranged from 1 to 8 with a mean of 4.1 (± 1.54 SD; suggesting up to four loci; Fig. 2), and we found between 1 and 6 supertypes per individual, with a mean of 3.07 (± 1.01 SD). However, only one individual from Lushoto carried 6 distinct supertypes (Fig. S3). The number of private alleles varied between study sites, ranging from 10 in Mvomero to 22 in Lushoto. All supertypes were present across all sites. This suggests that functional diversity is maintained across geographically distant sampling populations. Nevertheless, the ANOSIM identified that allele and supertype composition differed significantly between study sites (Fig. 2; *R* = 0.178; *p* = 0.001 and Fig. S3; *R* = 0.118, *p* = 0.001, respectively) and, thus, the subsequent co-occurrence analysis was computed separately for each site.

### Effects of MHC diversity on *Y. pestis* infection and flea burden

**Fig. 3:**
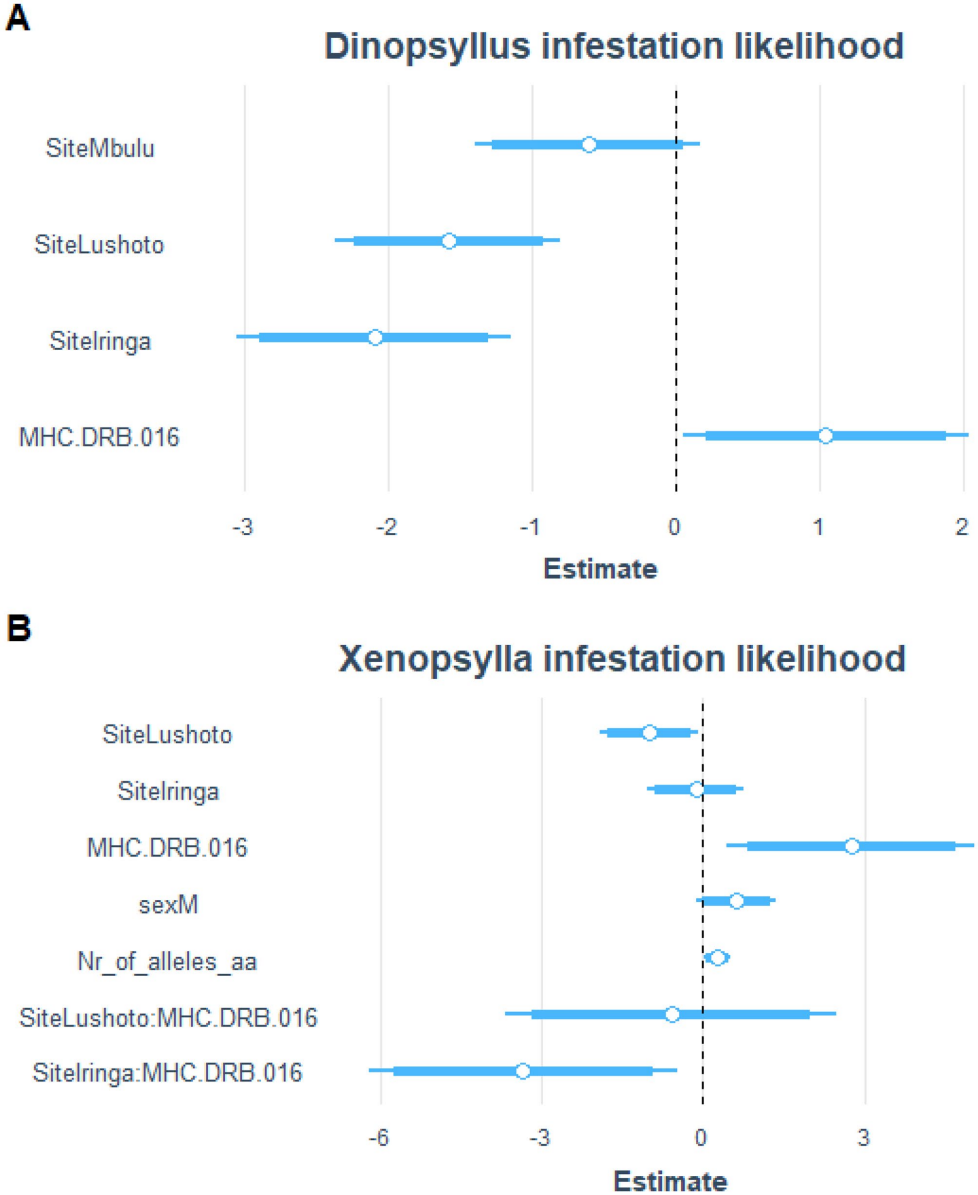
Model averaged parameter estimates and their 95% confidence intervals of GLMs examining the effect of MHC class II *DRB* allele DRB*016, sex, number of MHC alleles and study site on *Dinopsyllus* (**A**) and *Xenopsylla* (**B**) infestation on *M. natalensis*

Given our limited replication, we were unable to detect an association between individual flea burden and *Y. pestis*-positive iELISA results. While the co-occurrence analysis suggested an association between MHC II DRB*014 and *Y. pestis*-positive iELISA in Mvomero (Fig. S4), this effect was not supported by a GLM across sites since removing allele DRB*014 improved model fit according to our information theory approach. No MHC supertypes were linked to *Y. pestis*. But based on the co-occurrence model (Table 1) and the confirmatory GLMs, infestation likelihood with *Dinopsyllus* and *Xenopsylla* was positively associated with allele DRB*016 ((Fig. 3, (GLM, estimate: 1.05 ± 5.09, *p* = 0.039; GLM, estimate: 2.75 ± 1.18, *p* = 0.019, respectively)).

**Table 1:** MHC II DRB alleles positively associated with *Y. pestis* infection and flea infestation on *M. natalensis* individuals based on the co-occurrence analysis

	**Co-occurrence**	**Study site**	***P* value**
**MHC class II DRB allele**			
DRB*014	*Y. pestis*	Mvomero	0.046
DRB*014	*Dinopsyllus*	Iringa	0.015
DRB*016	*Dinopsyllus*	Iringa	0.048
DRB*004	*Dinopsyllus*	Lushoto	0.009
DRB*016	*Xenopsylla*	Mbulu	0.009
DRB*017	*Xenopsylla*	Mbulu	0.009
DRB*028	*Xenopsylla*	Mbulu	0.037
**Positively correlated alleles per site**			
DRB*004	DRB*014	Lushoto	
DRB*016	DRB*017	Mbulu

The best model to include an interaction between allele DRB*016 and study site on *Xenopsylla* presence (*p* < 0.001, Fig. 3): individuals carrying DRB*016 had a higher likelihood of *Xenopsylla* infestation in Mbulu, whereas in Iringa DRB*016 was associated with a lower infestation likelihood (GLM, estimate: − 3.35 ± 1.46, *p* = 0.022). Although the co-occurrence analysis suggested an association between MHC supertype 1 and *Xenopsylla* infestation likelihood, this was not supported in a confirmatory GLM (estimate: 0.15 ± 0.42, *p* = 0.716). As for the influence of other covariates on *Xenopsylla* infestation likelihood, the number of alleles had a weak effect (GLM, estimate: 0.26 ± 0.13, *p* = 0.042), and there was a lower likelihood of infestation in Lushoto (GLM, estimate: − 1.00 ± 0.47, *p* = 0.035). Sex had no effect (Fig. 3B).

## Discussion

Little is known about the true distribution of sylvatic plague in Tanzania—a bias inherent in many if not all studies of plague ecology in Tanzania that are only initiated after or during epidemics. Sylvatic hotspots without records of spillover to humans thus remain unidentified. Our study provides evidence that *Y. pestis* is still circulating in sylvatic reservoirs, such as *M. natalensis*, in endemic foci/areas (Iringa, Lushoto, Mbulu), but is also present in areas without historic records of human plague, such as Mvomero. A similar observation has been reported in south-western Zimbabwe, where a *Y. pestis*-positive *M. natalensis* individual was captured in a non-endemic area (Banda et al. 2022). Aside from *Y. pestis*, we recorded a high diversity of fleas in *M. natalensis* and an exceptional high diversity of MHC II alleles. Yet, we were unable to detect a clear link between *Y. pestis* infection and its vectors, or between host immunogenetic diversity and *Y. pestis* or common fleas. The only association were between the MHC class II allele DRB*016 and flea burden with either *Xenopsylla* or *Dinopsyllus*, and the direction of association differed between sites for *Xenopsylla.* Our work raises awareness of the potential of sylvatic reservoirs in spreading mammalian pathogens, such as the plague. At the same time, it emphasizes that exhaustive studies are needed, particularly in hosts with an exceptional immunogenetic diversity, to understand local, regional, and pancontinental differences (e.g., in the case of other reservoirs such as *Rattus rattus*) and similarities to reveal spatial differences in resistance.

We found a low prevalence of *Y. pestis* (9.8%), which is a common observation for *Y. pestis* in natural foci during inter-epizootic periods (Eisen and Gage 2009; Mahmoudi et al. 2021; Rahelinirina et al. 2022). This is especially true for enzootic hosts like *M. natalensis* that develop antibodies and survive the infection, but equally serve as a reservoir for the continuing transmission cycle, thus maintaining plague in endemic foci for decades (Kilonzo et al. 2005; Makundi et al. 2008; Ziwa et al. 2013b). The role of rodents as plague reservoirs in Tanzania is fairly well understood, and more than ten rodent species are hypothesized to serve as potential plague reservoirs (Msangi 1968; Kilonzo et al. 2005; Makundi et al. 2008; Kessy et al. 2023). Yet, the true reservoir of plague remains elusive, not at last due to the high number of potential reservoir species. Because *M. natalensis* is common, widespread, and peri-domestic, a role as potential plague reservoir is likely, even though they could merely be spillover hosts from an unknown cryptic reservoir as was reported to be the case in other species (Danforth et al. 2018; Colman et al. 2021).

Plague has rarely been examined in fleas in Tanzania during active phase of human plague epidemics or epizootics (Kilonzo and Mhina 1982; Makundi et al. 2008), and when examined during quiescent periods, it has not been detected (Haule 2013; Hang’ombe et al. 2014; Leulmi et al. 2014). This appears to be representative for flea-*pestis* patterns as has been found across various African countries (Bai et al. 2017; Rahelinirina et al. 2022) and the USA (Bron et al. 2019; Colman et al. 2021); although, there are exceptions (Hang’ombe et al. 2012; Ehlers et al. 2020). Regardless of data paucity on *Y. pestis* detection in fleas, *Dinopsyllus* and *Xenopsylla* species are the presumed key vectors involved in plague transmission in Tanzania because of their predominance in plague foci. We were, however, unable to detect a clear link between *Xenopsylla* or *Dinopsyllus* burden and individuals testing *Y. pestis*-positive. Plague foci in Tanzania differ in host diversity (Haikukutu et al. 2022; Kilonzo et al. 2005) and vector diversity (as shown in the current study). *Xenopsylla* was, for instance, not found in Mvomero, which presumably explains why human plague cases have never been reported in this area despite the presence of sylvatic plague. The two predominant fleas in plague foci in Tanzania plausibly play different roles in plague transmission owing to differences in host preference: *Dinopsyllus* might be involved in plague transmission between rodents, while *Xenopsylla* transmit plague from to rodents to human as it readily feed on humans when their natural hosts are not available. In fact, and unlike *Dinopsyllus*, only *Xenopsylla* species are considered efficient vectors of plague to humans (Zhang et al. 2015; Hinnebusch et al. 2017).

Only a limited number of studies investigated the immunogenetic basis of resistance against *Y. pestis* and flea infestation in wild rodents ((e.g., *C. gunnisoni*, *R. opimus*, *R. rattus* (Tollenaere et al. 2012; Cobble et al. 2016; Nilsson et al. 2018)). Likewise, the MHC of *M. natalensis* has rarely been investigated (Goüy de Bellocq and Leirs 2009, 2010), despite the rodents being a reservoir of some of the best-known pathogens like *Y. pestis* (Haikukutu et al. 2022) and Lassa virus (Olayemi and Fichet-Calvet 2020) and its pancontinental distribution. We report a total of 111 MHC class II DRB alleles and nine distinct MHC supertypes in *M. nataliensis* in four sites. While this is high in comparison with the 5–32 alleles found in shrews (e.g., Oliver and Piertney 2006; Scherman et al. 2014), the six found in a bottlenecked population of prairie dogs (Cobble et al. 2016), or the 27 identified in yellow-necked mice (*Apodemus flavicollis*; Meyer-Lucht and Sommer 2005), previous work on the MHC diversity in *M. nataliensis* caught only in Morogoro described 21 alleles in just 24 individuals (Goüy de Bellocq and Leirs 2010). Given that we sampled a higher number of individuals and across four geographically separate locations, we likely captured the species regional MHC diversity with many locally unique variants.

We were unable to detect a significant association between MHC class II alleles or supertypes and *Y. pestis* infection in *M. natalensis*. This, however, does not imply a lack of plague-mediated selection acting on the MHC but could possibly be due to low statistical power. In fact, the variation detected in the MHC allele composition between sites in the current study may imply geographic variations in pathogen-mediated selection, including by strains of *Y. pestis* (a complex yet to be disentangled in Tanzania) similar to reports from Madagascar (Vogler et al. 2011) and the many other pathogens likely to vary across time and space (Oliver et al. 2009). Moreover, several nucleotide sequences translated into the same amino acid allele, which possibly speaks to balancing selection conserving these sequences. Gene duplication plays an essential role in adaptation and cause important adaptive variation affecting the structural organization of the MHC molecules that bind and present antigens to the T-cells (Qurkhuli et al. 2019). Nilsson et al. (2018) reported that duplication of the MHC class II gene in plague-resistant great gerbil provides high peptide binding affinity for *Yersinia* epitopes. Similarly, Tollenaere et al. (2012) report gene duplication of MHC class I linked *PSD4* loci in the plague-resistant *R. rattus* in Madagascar. Work from European badgers suggests that plague resistance is indeed a polygenic trait with not just one gene determining survival (Sin et al. 2014). This is not far-fetched given the compelling evidence that the Black death pandemic–shaped human immunity, favoring individuals with more copies of the selectively advantageous haplotype (Immel et al. 2021; Klunk et al. 2022). *Y. pestis* may well exhibit a strong selective pressure conserving the duplicated sequences in *M. natalensis*; however, due to low *Y. pestis* prevalence and the exceptionally diverse DRB region of the MHC with many local variants, we did not find support for a strong link.

Despite the lack of a clear link between *Y. pestis* and MHC, we found evidence that MHC II allele DRB*016 was associated with flea burden in *M. natalensis*. For *Xenopsylla*, the effect of DRB*016 varied between sampling sites, which lends weight to the suggestion of local adaptation owing to locally distinct pathogen-mediated selection (Oliver et al. 2009). In another study, the MHC allele *Rhpu*-DRB*1 was associated with high parasite load in the four-striped grass mouse (*Rhabdomys pumilio*, Froeschke and Sommer 2005). Ectoparasites downregulate host innate and specific acquired immune defenses through special mechanisms that involve salivary peptides derived from anticoagulants, antiplatelets, vasodilators, and immunomodulators, which are then presented to antigen-specific T-lymphocytes at ectoparasite attachment sites by specific host MHC class II molecules (Rechav 1982). This triggers an immune cascade, which include specific antibody response, interfering with ectoparasite attachment and nutrient absorption by deactivating saliva mediated proteins that play key roles in pathways geared towards overcoming ectoparasite infestation. Immunological defense against ectoparasites is costly, a trade-off between the cost of reproduction and immune defense against ectoparasites in bats has previously been reported (Schad et al. 2012). Thus far, no fitness trade-offs were uncovered in *M. natalensis*, but a distinction between susceptible and resistant individuals might prove meaningful (Schmid et al. 2023). It is worth noting that ectoparasite abundance is a multifactorial trait influenced by environmental factors (e.g., temperature and humidity) and host characteristics such as home range, social system, sex, reproductive state, age, and body size (Maaz et al. 2018; López-Pérez et al. 2022), and, thus, longitudinal studies (e.g., capture-mark-recapture) encompassing all these factors are needed to paint a better picture of the interacting factors.

Clustering of functional MHC alleles into supertypes was suggested as remedy to overcome the lack of statistical power to detect pathogen-mediated selection on many nucleotide or amino acid alleles with great similarity, particularly in strongly spatially structured but geographically wide-ranging hosts (Herdegen-Radwan et al. 2021). However, we found no link between *Y. pestis* or flea vectors and MHC supertypes. Since MHC molecules encoded by distinct alleles may differ slightly in the range of bound peptides, which leads to variation in functionality despite similar peptide motifs (Kaufman 2020), the lack of association could suggest suboptimal supertyping that was unable to capture meaningful single amino acid differences and thus merged functionally still too distinct alleles into the same supertypes. However, because we also only found a single MHC allele associated with fleas and none with *Y. pestis*, we suspect our sample size is still insufficient to detect pathogen/vector and MHC allele/supertype associations (Gaigher et al. 2019).

Resources for surveillance in Tanzania as in other African countries are limited and rather directed at diseases of greater concern (Jowett and Miller 2005). But we have shown here that even sporadically occurring pathogens, like *Y. pestis*, and diseases of low incidence rates in humans, such as plague, quiescently circulate through sylvatic hosts or vectors. Our work is particularly timely given the recent spillover to humans reported in Babati district near Mbulu (Mwalimu et al. 2022). Even if surveillance was regular, endemic foci are often preferentially surveyed, though we have detected *Y. pestis*-positive in an area without records of human plague. Therefore, our work stresses the importance of regularly monitoring wildlife hosts and reservoirs throughout their range.

## Conclusion

Investigating a disease in natural populations is complicated, even more so if the disease has latent patterns of transmission and maintenance like plague. In this study, we characterized the MHC class II diversity of an important plague reservoir to gain new insight into the genetic background of plague persistence in Tanzania. We found a single MHC class II allele associated with plague vectors. Longitudinal studies to generate plague surveillance data coupled with studies encompassing whole genome or targeted re-sequencing and experimental infections would propel our understanding plague persistence in sylvatic reservoirs.

### Supplementary Information

Below is the link to the electronic supplementary material.

Supplementary file1 (DOCX 541 KB)

## Data Availability

MHC-DRB allele sequences and code are available on Github (https://github.com/LHaikukutu).
