# Supplementary material for: Immunogenetics, sylvatic plague and its vectors: insights from the pathogen reservoir Mastomys natalensis in Tanzania
Source: Immunogenetics. 2023 Oct 19;75(6):517–30. doi: 10.1007/s00251-023-01323-7 (PMC10651713; doi:10.1007/s00251-023-01323-7)
Supplement: Supplementary file 1 — Supplementary file1 (DOCX 541 KB) [file 251_2023_1323_MOESM1_ESM.docx]

**Supplementary material**

**Supplementary Tables**

**Table S1:** Positive site-specific selection identified with CodeML models, FUBAR, SLAC and MEME.

| **Method** | **Log(L)** | **Positively selected sites (PSS)** |
| --- | --- | --- |
| CodeML M1 | -3605.73 | Not allowed |
| CodeML M2 | -3550.45 | 5, 7, 13,16, 35, 36, 46,49, 50, 53, 57 |
| CodeML M7 | -3596.07 | Not allowed |
| CodeML M8 | -3543.70 | 5, 7, 13,16, 35, 36, 46,49, 50, 53, 57 |
| FEL | -3691.38 | 5, 50 |
| MEME | -3743.57 | 3, 24, 36, 46, 50, 51, 56, 57 |
| SLAC | -3743.57 | 5,13, 17, 46, 50, 57 |
| FUBAR | -3743.57 | 5 |
| **Summary** |  | **5, 3, 7, 13, 16, 24, 35, 36, 46, 49, 50, 53, 56, 57** |

**Table S2:** MHC-DRB allele assignment into nine supertypes (ST).

**Table S3:** Results of a generalized linear model with the common fleas *Dinopsyllus* and *Xenopsylla* as a dependent variable, and study sites as predictors.

| **Flea (genus)** | **Site comparisons** | | **Estimate** | **Std. Error** | **z value** | **Pr(>\|z\|)** |
| --- | --- | --- | --- | --- | --- | --- |
| ***Dinopsyllus*** |  | (Intercept) | -1.87 | 0.34 | -5.51 | < 0.001* |
|  | Iringa | Lushoto | 0.35 | 0.42 | 0.85 | 0.40 |
|  |  | Mbulu | 1.34 | 0.42 | 3.21 | < 0.001* |
|  |  | Mvomero | 1.87 | 0.46 | 4.03 | < 0.001* |
|  | Lushoto | (Intercept) | -1.52 | 0.24 | -6.31 | < 0.001* |
|  |  | Iringa | -0.35 | 0.42 | -0.85 | 0.40 |
|  |  | Mbulu | 0.99 | 0.34 | 2.89 | < 0.001* |
|  |  | Mvomero | 1.52 | 0.40 | 3.82 | < 0.001* |
|  | Mbulu | (Intercept) | -0.53 | 0.24 | -2.20 | 0.0280 * |
|  |  | Lushoto | -0.99 | 0.34 | -2.89 | < 0.001* |
|  |  | Iringa | -1.34 | 0.42 | -3.21 | 0.00133 * |
|  |  | Mvomero | 0.53 | 0.40 | 1.34 | 0.18 |
|  | Mvomero | (Intercept) | 0.00 | 0.32 | 0.00 | 1.00 |
|  |  | Mbulu | -0.53 | 0.40 | -1.34 | 0.18 |
|  |  | Lushoto | -1.52 | 0.40 | -3.82 | < 0.001* |
|  |  | Iringa | -1.87 | 0.46 | -4.03 | < 0.001* |
| **Xenopsylla** | Iringa | (Intercept) | -18.57 | 1031.32 | -0.02 | 0.99 |
|  |  | Mbulu | 17.37 | 1031.32 | 0.02 | 0.99 |
|  |  | Lushoto | 16.40 | 1031.32 | 0.02 | 0.99 |
|  | Lushoto | (Intercept) | -2.17 | 0.30 | -7.12 | < 0.001* |
|  |  | Mbulu | 0.98 | 0.41 | 2.37 | 0.0177 * |
|  |  | Iringa | 0.61 | 0.43 | 1.41 | 0.16 |
|  | Mbulu | (Intercept) | -1.19 | 0.28 | -4.31 | < 0.001* |
|  |  | Lushoto | -0.98 | 0.41 | -2.37 | 0.0177 * |
|  |  | Iringa | -0.37 | 0.41 | -0.90 | 0.37 |

* Significant at 0.05

**Suplementary Figures**

**Figure S1**


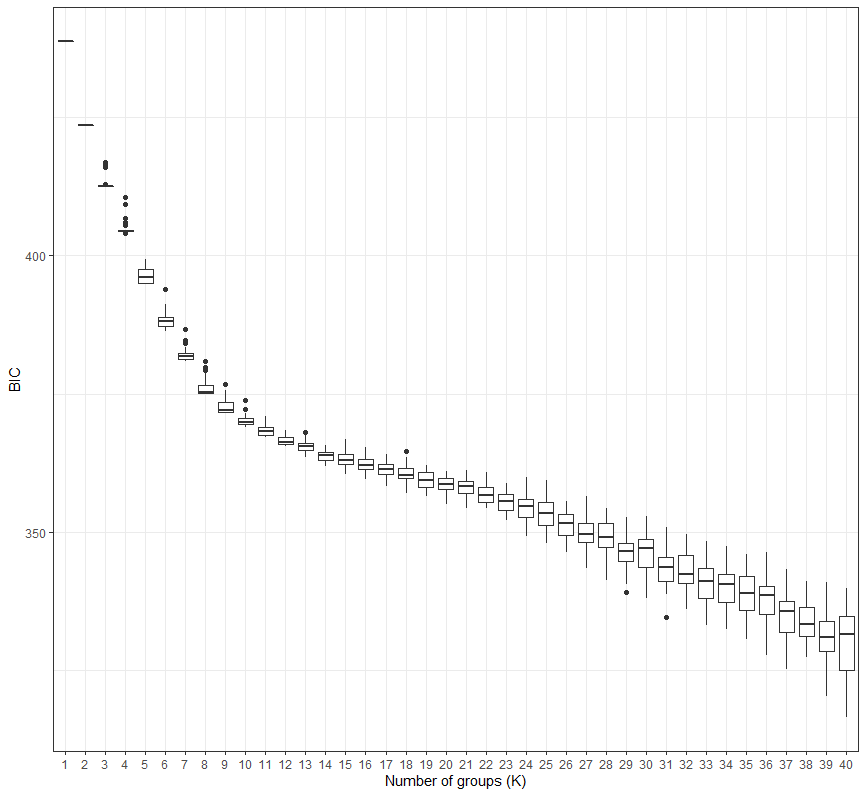


**Figure S1:** The BIC curve of DAPC clustering shows an optimum of nine supertypes.


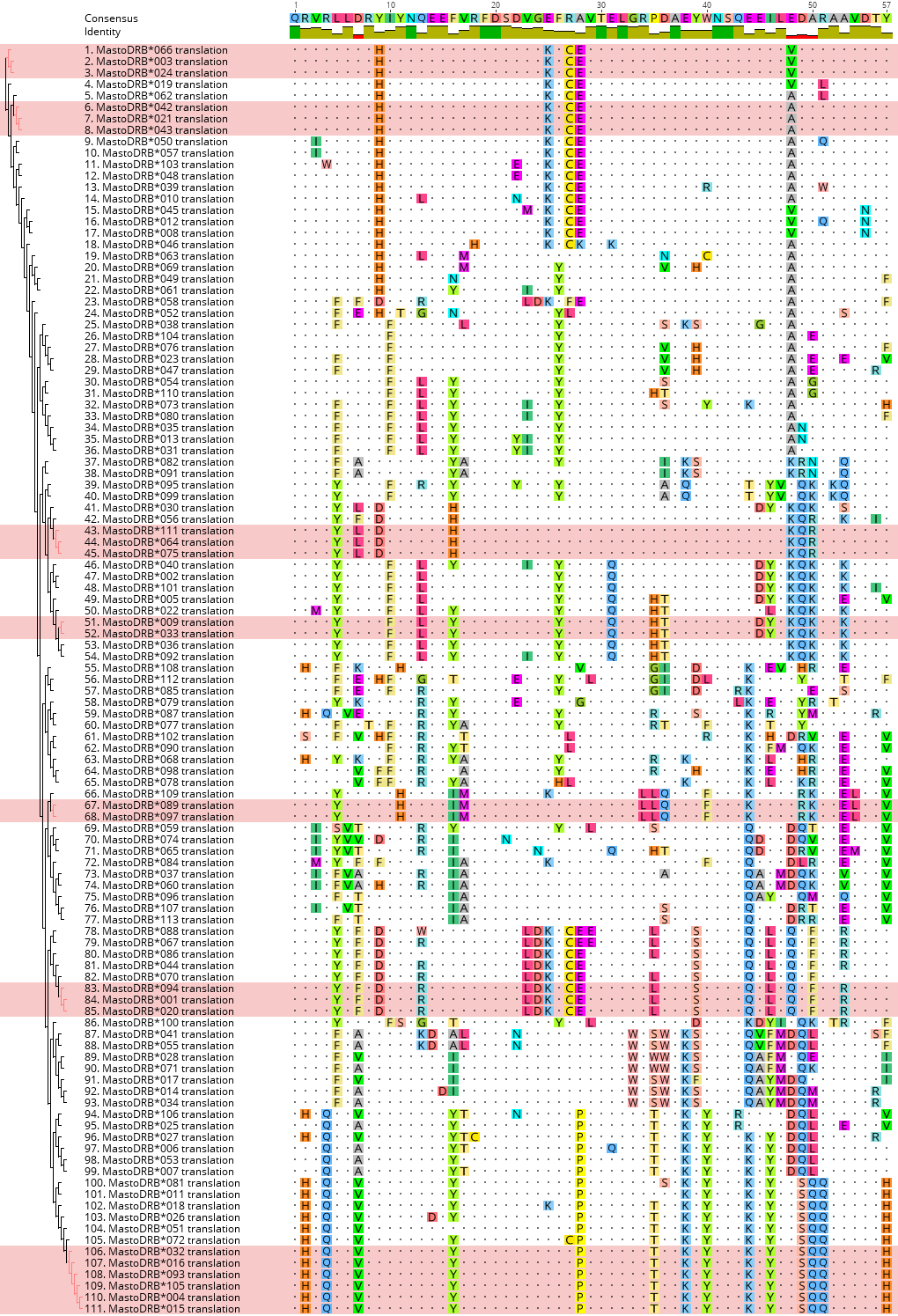


**Figure S2:** Translation of MHC class II nucleotide into amino acid alleles of *M. natalensis.* Duplicated amino acid alleles are highlighted in pink. The Tree was calculated in Geneious 11.1.5 using Jukes-Cantor as the genetic distance model and UPGMA as Tree build method, with bootstrapping to randomize (10.000 repeats) using Geneious default settings.


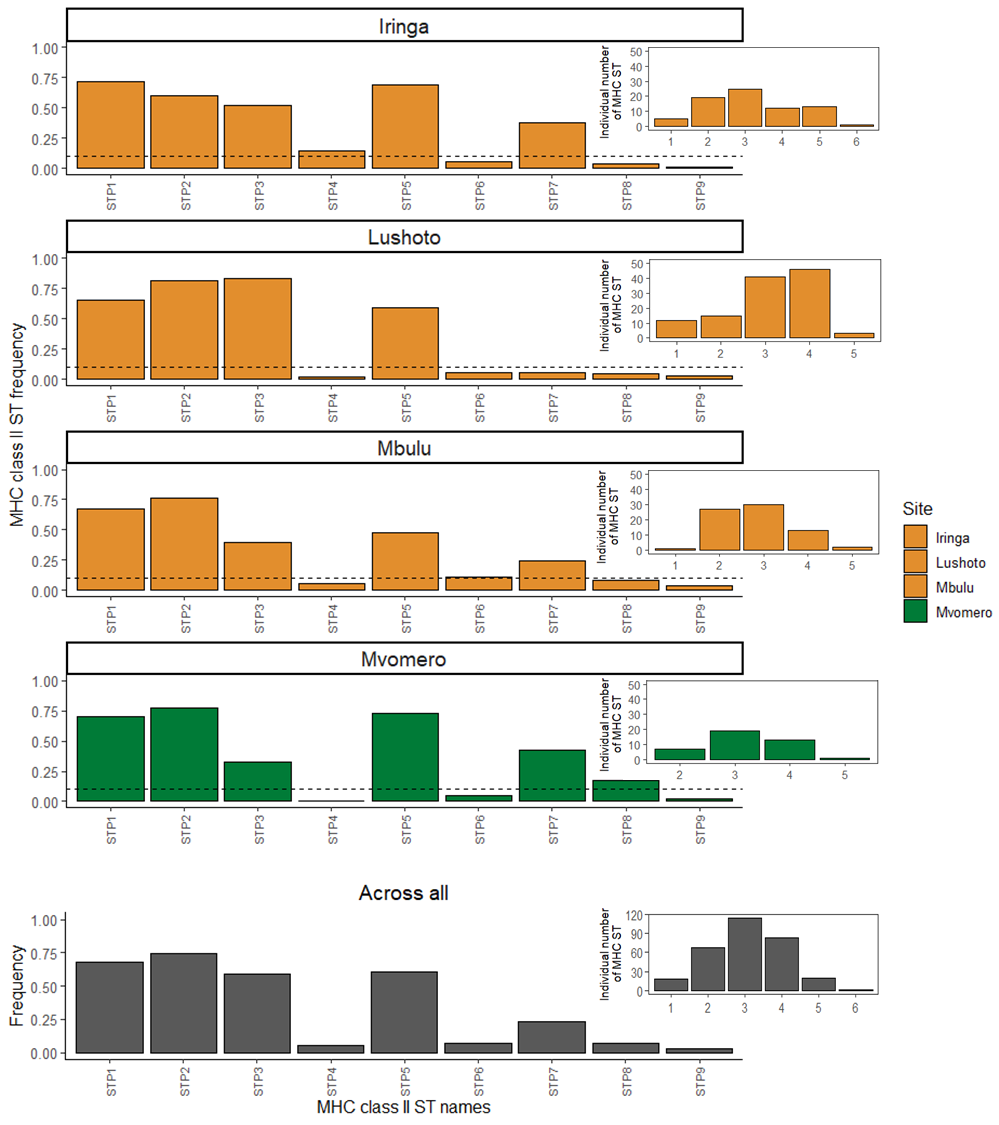


**Figure S3:** The frequency of MHC supertypes and total number of supertypes (per individual among 305 M. natalensis genotyped) per site and across all sampling sites. Orange shows districts considered to be plague endemic i.e., sites with a history of human plague, green indicates a non-plague district, i.e., site with no history of human plague. The dashed line indicates the 10% threshold for supertypes to be included in the co-occurrence analysis.


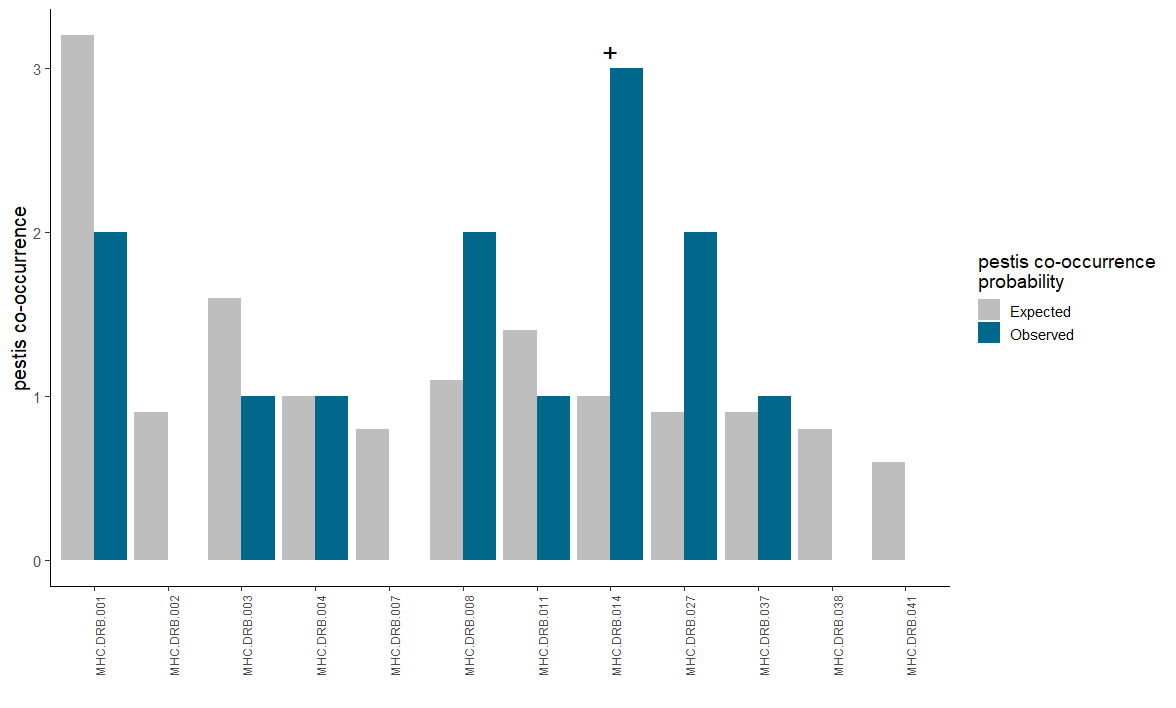


**Figure S4:** Association of MHC alleles with *Y. pestis* co-occurrence in Mvomero. A cross (+) indicates significant differences between expected and observed values.
